# Exploring implementation processes of a parent-targeted educational video for improving newborn pain treatment: A sequential exploratory mixed-methods study

**DOI:** 10.1177/13674935231176888

**Published:** 2023-06-23

**Authors:** Catherine Larocque, Carolina Lavin Venegas, Sandra Dunn, Marsha Campbell-Yeo, Lucy Gilmore, JoAnn Harrold, Jiale Hu, Leanne McArthur, Shokoufeh Modanloo, Stuart G Nicholls, Pat O’Flaherty, Shahirose Sadrudin Premji, Jessica Reszel, Sonia Semenic, Janet E Squires, Bonnie Stevens, Monica Taljaard, Marie-Josee Trepanier, Kathy Venter, Jodi Wilding, Denise Harrison

**Affiliations:** 1 6363 University of Ottawa, Ottawa, Ontario, Canada; 2 574118 Better Outcome Registry and Network, Ottawa, Ontario, Canada; 3 27338 Children’s Hospital of Eastern Ontario Research Institute, Ottawa, Ontario, Canada; 4 3688 Dalhousie University, Halifax, Nova Scotia, Canada; 5 IWK Health Centre, Halifax, Nova Scotia, Canada; 6 60423 Headwaters Health Care Centre, Orangeville, Ontario, Canada; 7 27338 Children’s Hospital of Eastern Ontario, Ottawa, Ontario, Canada; 8 10055 The Ottawa Hospital, Ottawa, Ontario, Canada; 9 6889 Virginia Commonwealth University, Richmond, Virginia, United States of America; 10 10033 London Health Sciences Centre, London, Ontario, Canada; 11 10055 Arthur Labatt Family School of Nursing, London, Ontario, Canada; 12 10055 Ottawa Hospital Research Institute, Ottawa, Ontario, Canada; 13 Champlain Maternal Newborn Regional Program, Ottawa, Ontario, Canada; 14 Queen's University, Kingston, Ontario, Canada; 15 5620 McGIll University, Montréal, Canada; 16 27338 The Hospital for Sick Children, Toronto, Ontario, Canada; 17 483367 The University of Toronto, Toronto, Ontario, Canada; 18 School of Epidemiology and Public Health, University of Ottawa, Ottawa, Ontario, Canada; 19 71545 Baby-Friendly Initiative Ontario, Toronto, Ontario, Canada; 20 University of Melbourne, Parkville, Victoria, Australia; 21 Murdoch Children’s Research Institute, Melbourne, Victoria, Australia

**Keywords:** Breast feeding, kangaroo care, newborn infant, sucrose, procedural pain, neonatal screening, translational science implementation science

## Abstract

Despite known analgesic effects of breastfeeding (BF), skin-to-skin care (SSC), and sweet solutions (sucrose) for newborns, these interventions remain underutilized. Our team produced a five-minute parent-targeted video (BSweet2Babies) demonstrating BF, SSC, and sucrose during newborn blood sampling. We conducted a sequential exploratory mixed-methods study with eight maternal-newborn units across Ontario, Canada to identify barriers and facilitators to implementing the video and the three pain management strategies.

Over a 6-month period, data collection included 15 telephone interviews, two email communications, and three community of practice teleconferences with the participating sites (*n* = 8). We used the Theoretical Domains Framework as the coding matrix. Participants discussed integrating the video in prenatal education and the importance of involving leadership when planning for practice change. Key barriers included lack of comfort with parental presence, perception of high complexity of the strategies, short postpartum stays, competing priorities, and interprofessional challenges. Key facilitators included alignment with the Baby-Friendly Hospital Initiative, modeling by Lactation Consultants, and frequent reminders.

## Background

Almost all newborns have blood sampling for newborn screening (NBS) and preterm and sick babies have multiple painful procedures during their hospitalization (Cruz et al., 2016). Blood sampling is painful and, for sick and preterm babies, repeated painful procedures increase risk of adverse long-term neurobehavioral outcomes (Duerden et al., 2017; Valeri et al., 2015; Walker, 2019). High quality synthesized evidence supports analgesic effects of breastfeeding (BF) (Benoit et al., 2017; Shah et al., 2012), skin-to-skin care (SSC) (Johnston et al., 2017), and sweet solutions (Harrison et al., 2017a; Stevens et al., 2016a) during blood sampling, and professional associations worldwide recommend the use of these pain management strategies (Balice-Bourgois et al., 2020; Lee et al., 2014; Newborn Screening Ontario, 2018).

Education about pain care for newborns has primarily targeted healthcare providers (HCPs), yet studies continue to show inconsistent use of the evidence (Courtois et al., 2016; Cruz et al., 2016; Orovec et al., 2019). In a systematic review of 81 trials, Forsetlund et al., (2009) found educational meetings were ineffective at changing clinician behaviors, particularly when outcomes were not considered to have serious consequences. However, patient-mediated knowledge translation (KT) interventions may be effective in changing clinician behavior (Stacey and Hill, 2013). In the context of neonatal pain, parents want to be involved in comforting their baby during painful procedures (Franck et al., 2011; Franck et al., 2012; McNair et al., 2020), and neonates are more likely to receive pain treatment if parents advocate for it (Johnston et al., 2011). Therefore, KT interventions targeted at both parents and HCPs may reduce this knowledge-practice gap (Benoit et al., 2016).

## Study aim

The aim of this study was to identify barriers and facilitators to the implementation of the Be Sweet to Babies (BSweet2Babies) video and to the use of BF, SSC, and sucrose for pain management during NBS in clinical settings.

## Methods

### Be Sweet to Babies video (BSweet2Babies)

Our research team produced a brief five-minute parent-targeted and mediated video titled Be Sweet to Babies (BSweet2Babies) (https://youtu.be/L43y0H6XEH4). This video portrays BF, SSC, and sucrose during heel lance and venipuncture with voice-over explaining how parents can help their babies by partnering with clinicians to use these strategies. This video is parent-targeted because it engages parents to improve their knowledge and is parent-mediated in its goal to change HCPs behavior through the parent-provider relationship (Stacey and Hill, 2013). Pilot testing of the video with HCPs (Harrison, et al., 2017b) and parents (Korki De Candido et al., 2020; Lavin Venegas, et al., 2019a) demonstrated the video was acceptable, persuasive, and feasible to show to parents.

### Study design

We designed this study as a process evaluation. Due to challenges with recruitment, we altered the study design to a sequential exploratory mixed-methods design, in which the qualitative stage occurs before the quantitative stage and results are integrated after the completion of both phases (Pluye and Hong, 2014). In this manuscript, we report on the first phase of the multiphase study, understanding barriers and facilitators to implementing the video and pain management strategies.

### Ethical approvals

This study is registered at ClinicalTrials.gov (NCT03099252). After obtaining ethical approval from the PI’s host institution (The Children’s Hospital of Eastern Ontario), the study was submitted to Clinical Trials Ontario (CTO). CTO provides a streamlined approach for ethical approval of multi-center trials in the province of Ontario, Canada. Following approval from CTO (Project#0832), each of the participating sites also provided ethical approval.

### Sampling and inclusion criteria

Hospital units providing inpatient maternal/newborn care across Ontario were recruited through convenience sampling. Hospitals were eligible for inclusion if they (1) provided Level 1 or Level 2 maternal/newborn care (Provincial Council for Maternal and Child Health, 2023); (2) had a birth volume of >50 per year; (3) had ≤85% use of pain management strategies (BF, SSC, sucrose) during NBS, as per baseline data (Better Outcome Registry and Network (BORN) information system (BIS)) (BORN, 2019a); (4) had ≤50% missing pain management data element in the BIS. Forty-seven of 86 (55%) units providing maternal/newborn care in Ontario (level 1, 2) were eligible for inclusion and invited to participate via email from the research coordinators (JR and JW) with three reminder emails sent at weekly intervals (four attempts to contact over 4 weeks). The coordinators contacted nurse managers or their delegates who had expressed interest, and if recruited, the study was launched at these sites following ethical approval from their institution. We excluded pilot sites from the study sample.

### Implementation procedures

Participating sites received the following KT tools via their unit manager:

1. Parent-targeted BSweet2Babies video in 10 languages (English, French, Arabic, German, Hindi, Inuktitut, Mandarin, Parsi, Portuguese, Spanish) preloaded to an electronic tablet.
2. Parent cards (Supplemental File 1)—visual reminder for parents with a Quick Response (QR) code to scan directing them to the YouTube video.
3. BSweet2Babies poster (Supplemental File 2)—visual reminder for parents and HCPs.

**Figure 1.**
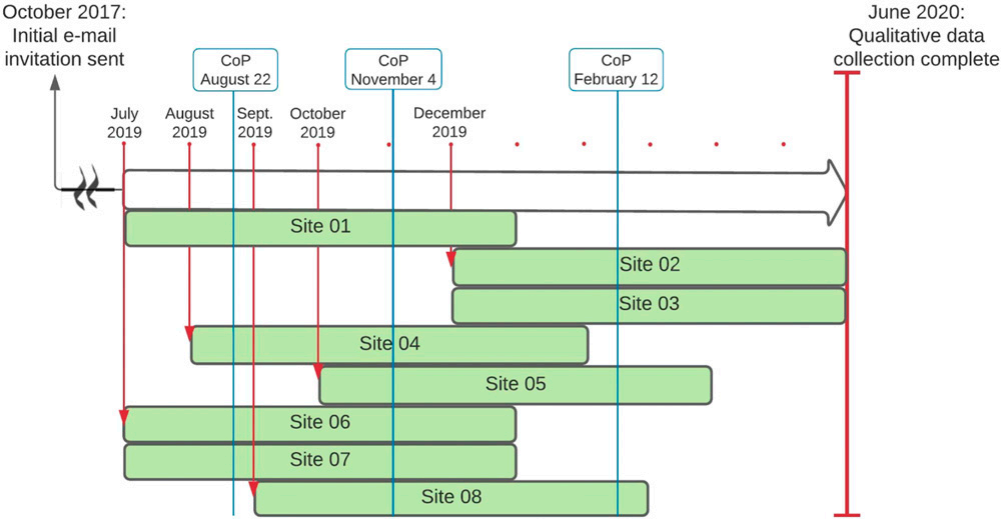
Study flow. *Note.* The first contact was made with eligible sites in October 2017. The first sites (i.e., 01, 06, 07) began their 6-month intervention period in July 2019. Rectangles labeled by site represent the beginning and end of each participating unit’s 6-month intervention period.

Using these tools and contacts with the research team (described below), sites implemented the video (viewing by parents) before NBS, and facilitated BF, SSC, or administered sucrose during NBS over a 6-month intervention period. See Figure 1 for the study flow. All processes and KT tools were piloted at two hospitals considered representative of the eligible units. With their local interdisciplinary teams, nursing leaders from both pilot sites advised on all aspects of the implementation process and delivery of the video, optimizing implementation fidelity.

### Data collection

Over the 6-month intervention period, the research team offered monthly one-on-one semi-structured telephone interviews with the nurse leaders at participating sites and bimonthly Community of Practice (CoP) Teleconferences with all participating sites. Interviews and CoP teleconferences were audio-recorded with consent and the interviewers (JW or CL) took field notes. Interviews and CoPs were transcribed verbatim, de-identified, and imported into QSR International’s NVIVO 12 for data management (QSR International Pty Ltd, 2018).

1. *Monthly Nurse Leader Phone Calls*

We used a semi-structured interview guide (Supplementary File 3) to explore barriers and facilitators to video implementation and use of pain management. Our research team pre-tested the interview guide at two pilot sites. All interviews except two were conducted by phone. Two participants requested contacts via email and the interview guide was emailed as requested to complete and return to coordinator JW.

2. *Bimonthly Community of Practice Teleconferences (CoPs)*

A CoP can be defined as:

“*groups of people who share a concern or a passion for something they do and learn how to do it better as they interact regularly*.” (Wenger, 2011: 1).

Therefore, the CoP teleconferences in this study were unstructured opportunities for discussion of barriers and facilitators to video implementation and use of pain management, to share strategies that worked or not, and strategies used to overcome barriers.

Following completion of data collection and transcript verification, all recordings were permanently deleted according to the PI’s host institution’s research ethics guidelines.

### Data analysis

Following each site’s completion of the 6-month intervention period, all transcripts (i.e., telephone interviews and CoPs) were analyzed using directed content analysis (Atkins et al., 2017). The Theoretical Domains Framework (TDF) informs implementation by understanding barriers and facilitators to the use of evidence (Atkins et al., 2017). Version 2 of the TDF (TDFv2) (Cane et al., 2012), consisting of 14 domains and 83 constructs, was used as the coding matrix. However, the directed (deductive) approach did not preclude an inductive analysis of the data to identify salient barriers and facilitators not captured in the TDF framework (McGowan et al., 2020). During data analysis, the domain “nature of the behavior,” which is found in version 1 (TDFv1) (Atkins et al., 2017) of the framework, emerged as a theme and was therefore included in our framework. We used the action, actor, context, target, and time (AACTT) framework to specify behaviors and ensure barriers and facilitators pertained directly to actions and actors of interest (Presseau et al., 2019) (see Table 1).

**Table 1.** Action, Actor, Context, Target, Time statement.

AACTT domain	Definition^†^	Video	Pain management
Action	A discrete observable behavior	Showing the video (or the parents viewing the video independently)	Implementing evidence-based practices of BF, SSC, or sucrose administration during newborn screening
Actor	The individual or group of individuals who performs (or should/could) the action	The nurse showing the video	The nurse performing the procedure
Context	The physical, emotional or social setting in which the actor performs (or should/could) the action	Within the participating site	
Target	The individual or group of individuals for/which/on behalf of whom the actor performs the action	Parent who will be watching the video	Baby who will be receiving pain management
Time	The time period and duration that the actor performs the action in the context with/for the target	1) During the 6-month intervention period 2) Showing the video to parents before the NBS	1) During the 6-month intervention period 2) Using BF, SSC, or sucrose for pain management during the NBS

† Definitions from: (Presseau et al., 2019).

There were two unique AACTT statements (Table 1), reflecting the dual inquiry of barriers and facilitators to showing the video, as well as the barriers and facilitators to the use of BF, SSC, or sucrose during NBS.

The analysis process occurred in four steps:

1. CL and CLV independently read and reread transcripts independently and data were deductively coded into the 14 TDFv2 domains and one TDFv1domain, as described above. Differences were reconciled through discussion. A data dictionary was developed to delineate each domain in the context of this study and reduce any potential overlap between domains.
2. Following data dictionary development, CL and CLV conducted a second review of the transcripts to ensure the same definitions were applied as the data dictionary evolved.
3. Following the second review of the transcripts, CL and CLV further organized coded data within each domain as either a barrier or facilitator (when possible) to behaviors specified in the AACTT framework (Table 2).
4. Authors DH and SD independently reviewed these findings and broader themes spanning multiple domains were extrapolated from the data.

**Table 2.** Telephone interview contacts by site.

Site	Completed contacts	Missed contacts	Could not reach participant	Declined by participant
01	M4: 7 November 2019 M5: 13 December 2019	M6: 7 January 2020	—	M1: 3 August 2019 M2: 3 September 2019 M3: 3 October 2019
02	M3: 3 February 2020	M1: 1 Dec 2020 M2: 1 Jan 2020	M4: 3 March 2020 M5: 3 April 2020 M6: 3 May 2020	—
03	M1: 20 January 2020	—	M2: 20 February 2020 M3: 20 March 2020 M4: 20 April 2020 M5: 20 May 2020 M6: 20 June 2020	—
04	M1: 24 August 2019 M3: 24 October 2019 M5: 17 December 2019	M2: 2 September 2019 M4: 2 November 2019 M6: 2 January 2020	—	—
05	M2: 15 November 2019 M3: 2 January 2020	M4: 18 January 2020	M5: 18 February 2020 M6: 18 March 2020	M1: 18 October 2019
06	M2: 9 September 2019 M4: 29 November 2019 M6: 23 January 2020^†^	M3: 11 October 2019 M5: 11 December 2019	—	M1: 11 August 2019
07	M1: 30 July 2019 M2: 3 September 2019^†^ M6: 6 January 2020	M3: 2 October 2019 M4: 2 November 2019 M5: 2 December, 2019	—	—
08	M2: 7 November 2019 M3: 13 December 2019	M4: 7 January 2020 M5: 7 February 2020	M6: 7 March 2020	M1: 3 October 2019
Total	*N* = 17	*N* = 14	*N* = 11	*N* = 6

† Contact conducted via email at the request of the participant.

*Note:* M designates month; M1 means first month of the intervention period, M2 the second month, etc.

## Findings

**Figure 2.**
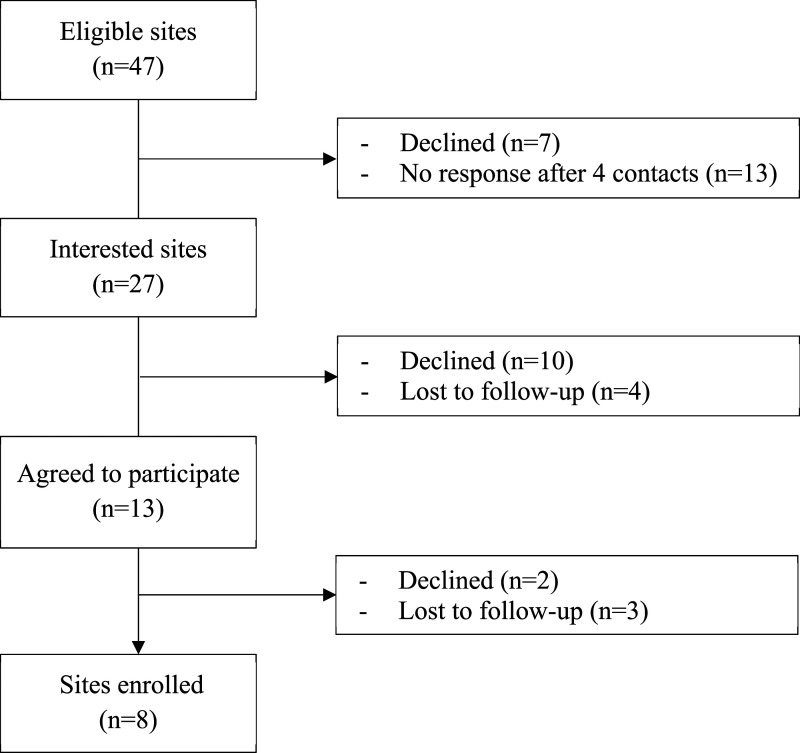
Consolidated Standards of Reporting Trials (CONSORT) flow diagram.

Between July 2019 and April 2020, nursing leaders from eight of 47 eligible units (17%) provided proxy consent to participate (Weijer et al., 2012), and completed the 6-month intervention (Figure 2). Four units provided level I maternal/newborn care, and four units provided level II maternal/newborn care. Birth volumes ranged from 501 to >2500 per year. Designated nursing leaders included Clinical Nurse Specialists (*n* = 2; 25%), unit managers (*n* = 3; 37%), charge or staff nurses (*n* = 2; 25%), and a Director of Maternal-Child and Inpatient Services (*n* = 1; 13%). While monthly interviews and bimonthly CoP teleconferences were offered, we completed 17 individual interviews (including two responses by email) and three CoP teleconferences, ranging from 20 to 60 min (see Table 2 and Figure 1, respectively).

Participants reported that most nurses already used, and continued to use, sucrose rather than BF or SSC and that, despite providing the video and education to parents, it was usually the nurse who initiated the conversation about pain management during NBS. All TDF domains were evident and identified, except emotion (Atkins et al., 2017, p. 4), and three key inductive themes emerged, namely, leadership, parent advocacy, and lab technicians. The following presents the most frequently coded TDF domains first, followed by three inductive themes. A detailed overview of barriers, facilitators, and illustrative quotes for each of the 15 TDF domains is summarized in Supplementary File 4.

### TDF domains

#### Environmental context and resources (TDFv2, domain 11)

All participants discussed barriers and facilitators related to environmental context and resources. Most participants reported that staff turnover and competing priorities, including implementation of electronic medical records or hospital renovations, were barriers to showing parents the video and using the strategies during NBS. Participants stressed the short length of postpartum hospital stay (generally 24 h after vaginal birth, and 48 h post-Caesarean section), necessitating intensive postnatal education within a short period. One participant reported postpartum families are already exhausted from labor and delivery and experiencing

“*information overload*” (Nursing manager of women and children’s health at site 05).

Considering the interplay between parental fatigue and heavy nursing workload, participants suggested delivering the parent video in the prenatal period, during preregistration clinics or prenatal classes.

The physical layout of units was also identified as both a barrier and facilitator. For many maternal/newborn units in Ontario, semi-private rooms are standard. Participants reported the space in semi-private rooms is limited, posing challenges to performing NBS with the baby BF or SSC, without disturbing other families.

“*The positioning is off. The room is too small. It’s tight. At night, it’s dark in the room. In a semi-private room, I’m having to turn on the lights and wake up the other patients*.” (Obstetrical Clinical Nurse Specialist at site 01)

While the small size of certain rooms can be a hindrance, participants described how units with a single-family-room design (labor, delivery, and postpartum care occur in the same room), facilitate showing the video and using the pain management strategies during NBS.

#### Nature of the behavior (TDFv1, domain 12)

Regarding the video, some participants reported it was brief, easy to deliver (via the study tablet or in-room televisions) and did not increase workload. Other participants reported their staff found showing the video to families awkward and more time-consuming, preferring to deliver the education face-to-face (or not at all). Another key challenge for showing the video reported was forgetfulness. Despite frequent reminders organized by the nursing leaders and provision of the KT tools, participants reported the nurses consistently forgot to show the video. For example:

*“… having to get the nurses to remember to do it and to take it as part of their job to do.”* (Nursing manager of women and children’s health at site 05).

Regarding the pain management strategies, the most frequently reported barriers included workload and time constraints, and difficulty changing

“*the way they’ve [the nurses] always done it*” (Participant in CoP 3).

Regarding the nature of the intervention itself (performing NBS with the infant BF or held SSC), our participants reported their staff found the strategies too complex.

#### Knowledge and Skills (TDFv2, domains one and two)

Almost all reported their staff felt uncomfortable performing blood sampling with the infant BF or held SSC. They mentioned staff concerns about physical positioning and ergonomics, a lack of comfort with the technique, and discomfort with parents observing the procedure. Even after the research team provided participating units with the HCP-targeted educational video demonstrating proper ergonomics for blood sampling during BF or SSC (Harrison, 2019), they continued to report their staff did not feel comfortable.

One participant reported having an International Board-Certified Lactation Consultant on staff provided opportunities for knowledge sharing and modeling, which ultimately led to an increase in staff performing blood sampling with the infant BF or SSC. At another site, a nurse leader reported they assigned a full-time nurse to lead the study and be responsible for showing parents the video, encouraging, and supporting the other nurses, and promoting the study. The nurse leader reported:

“*There was a quick pick-up from the nurses because she [assigned nurse] was communicating the information and teaching the nurses and because she’s a staff nurse like them, I think they actually bought into it. I saw the excitement.”* (Nurse manager at site 8).

### Inductive themes

#### Leadership

Leadership emerged within the domains of environmental context and resources, social influences, and intentions and goals. Participants discussed the importance of involving unit and organizational leadership in research and practice change. Two sites described how leadership buy-in and managers’ support were essential for ensuring consistent messaging to staff. The nurse leaders reported,

*“Our nurses took this seriously because it was coming from leadership […]. So, the nurses are taking this seriously because we are taking this seriously.”* (Obstetrical Clinical Nurse Specialist at site 01)

Further, initiatives and leadership from outside organizations exerted a positive social influence on nursing leaders. Six of the eight sites discussed how working towards their Baby-Friendly Hospital designation (World Health Organization and the United Nations Children's Fund (UNICEF), 2018; Baby-Friendly Intiative Strategy Ontario, 2014), made it easier for staff to integrate showing the video and using BF or SSC during NBS into their practice. Furthermore, participants discussed how reinforcement from the BIS (which provides sites with reports on their use of pain management)—being able to quantitatively see an increase in the proportion of infants receiving pain management during NBS—engendered professional confidence and enthusiasm amongst their staff. However, some participants mentioned that even with organizational leadership’s support for the study and the three pain management strategies, and despite external social influences, other priorities still prevailed.

#### Parent advocacy

Parent advocacy emerged as an inductive theme, falling within the domains of beliefs about consequences and social influences. Implicit in the goal of providing parents with the video is the assumption they will advocate for the use of pain management during their newborn’s blood sampling (McNair et al., 2020). All our participants discussed how the QR code on posters placed in patient rooms (Supplemental File 2) created an environment where parents could seek out the information about pain management on their own, both involving them in care and removing the burden from the nurse. As stated by one participant,

*“… we have had parents say, ‘Oh, I already watched it. I did it on my phone.’ So, the posters in the rooms are effective as well because we’ve gone to offer it to them, and they’ve already actually taken the initiative and gone on and watched it already.”* (Charge Nurse at site 06).

However, participants also discussed their perception that the unit nurses did not show parents the video or provide education about the three strategies because they did not want the parents to ask them to use one of the strategies, especially BF or SSC.

#### Lab technicians

Laboratory (lab) technicians and phlebotomists emerged inductively as key actors in the implementation of pain management during NBS. In some postpartum units, lab technicians perform NBS rather than the nurses. Participants described lab technicians’ concerns about ergonomics and the lack of clarity around their role description and scope of practice regarding pain management. To maximize the potential use of the strategies, some participants discussed plans to shift from lab technicians performing NBS to unit nurses. However, another participant discussed how, despite not initially involving the lab manager and staff, partnering with the lab leadership team to deliver an educational intervention was essential.

*“I just feel that it was the lab education that made the big difference. I feel like getting them onboard was a big win for us”* (Nurse manager at site 05).

## Discussion

Participants reported various barriers and facilitators to the implementation of the BSweet2Babies video and to the use of BF, SSC, and sucrose for pain management during NBS; they also described various strategies to address identified barriers. Most strategies reported by our participants were education products (e.g., posters) or reminders such as emails, notifications in the electronic record system, and paper cards affixed to care pathways. Participants also perceived more active approaches, such as unit champions, modeling and role-modeling, and, in one unit dedicating a full-time nurse to the study, as effective, although more resource-intensive. Consistent with other findings in the literature (e.g., Stevens et al., 2016b), participants reported interest, enthusiasm, and engagement with the project waned over time. According to Moore et al. (2017), understanding sustainability is a major challenge in implementation science. The findings of our study, namely, resource-intensity and waning enthusiasm underscore such challenges with feasibility, implementation, and sustainability of change.

### Barriers and facilitators to the implementation of the BSweet2Babies video

Barriers and facilitators reported by participants in our study regarding the BSweet2Babies video were contradictory. When nursing staff was tasked with facilitating parents to view the five-minute video, it was not always seen as practical, useful, or timely. Participants reported for some nurses, this was due to the perception they were already educating the parents and the video was more time-consuming than verbal education. This supports the findings of a systematic review by McNair et al. (2020), which found HCPs can act as gatekeepers who determine the flow of information to parents to maintain control over the situation. Our participants reported for some nurses, the BSweet2Babies video was seen as promoting parent-led practices which were not the nurses’ preferred practice. This preference of staff to exclude parents from newborn pain management has been previously reported (Hassankhani et al., 2020; Hu et al., 2020; Kyololo et al., 2014; McNair et al., 2020). Although parental involvement during NBS is just one example of evidence-based practice and family-centered care (FCC), the commonly reported barrier of excluding parents requires a larger cultural shift to truly embrace a FCC philosophy (Larocque et al., 2021).

Facilitators to showing parents the BSweeet2Babies video included the availability of a preloaded tablet; having posters with the QR code (Supplemental File 2); the physical presence of leadership on the unit (social influences); pre-existing knowledge and use of sucrose, breastfeeding, or SSC for pain treatment; having a supportive organization. Organizational leadership needs to ensure resources are in place to embrace parent partnership in care, which, in turn, requires priorities aligning with, and supporting, a FCC philosophy. Furthermore, some participants perceived providing education about NBS and pain management to parents in the prenatal setting was more effective because it removed the burden from nurses in the postpartum setting where families’ inpatient stays are short, and the workload is high.

### Barriers and facilitators to the use of BF, SSC, or sucrose during NBS

Participating nurse leaders believed that although unit nurses were knowledgeable about best practices for neonatal pain, consistent use of the strategies was challenging. This contradiction highlights that awareness and even understanding of the consequences was not enough to guarantee the desired action would occur. While the low-cost and effective pain management strategies of BF, SSC, or sucrose during NBS seem relatively simple at face value, participants reported a range of barriers.

As above, nursing leaders reported staff preferences to not involve parents in NBS. Similarly, a recently published survey showed BF or SSC were rarely used in a Canadian neonatal unit and parents were not present during any painful procedure (De Clifford-Faugère et al., 2019). Axelin et al. (2015) discuss nurses’ preference to not involve parents as a power imbalance with nurses acting as gatekeepers dictating parents’ level of involvement due to their perception that parents do not wish to be present during painful procedures. This perception conflicts with evidence that parents do wish to be present (Harrison, 2020; McNair et al., 2020). Whether nurses’ preferences to not involve parents in this study is due to perceived time restraints, environmental constraints, knowledge deficit, ergonomic issues, or power imbalances is unclear.

A further concerning barrier was that other priorities precluded, or took precedence over, pain management best practices. Our participants identified the introduction of electronic medical records, staff turnover, hospital renovations, and from March 2020 onward, the Coronavirus Disease 2019 (COVID-19) pandemic, as contributing to not using pain treatment for newborns. In the face of these organizational and environmental issues, implementing pain management best practices at an organizational and unit level was not a priority for some managers and nurses at the point of care. Modanloo et al., (2023) conducted a scoping review of pediatric research priorities and examined 30 articles where parents, youth, and families participated in the priority-setting process. Across these 30 articles they identified 455 priorities, and among these the most frequently reported was:

“*improving pain management and sedation practices*” (Modanloo et al., 2023: 13).

The contrast between the priorities reported by our participants and the priorities identified in Modanloo et al., (2023) highlights a dissonance between HCPs and families.

Another barrier was lack of time. The postnatal context is unique; the stay for most following vaginal birth is only 24 h, which is very little time to complete required tasks and education before discharge. Therefore, any additional education is a low priority. A study by Lavin Venegas, et al., (2019b), which involved showing parents of healthy newborns the same video used in this current study, found although parents preferred to use the parent-led interventions of BF and SSC, these were not subsequently used during NBS. In a follow-up study in the same postnatal setting, nurses reported similar barriers to those described by our participants in leadership roles (Lavin Venegas et al., 2019a), including perceived additional time required, concerns about the ergonomics of performing NBS while infants are held, and nurses preferring to remove newborns from the mother’s room for NBS. These findings, with the findings of this study, suggest that although providing education about NBS and pain management directly to parents allows them to advocate for their preferred pain management, the organizational and unit context, environmental setting, and nursing or phlebotomy staff must elicit and be supportive of parents’ wishes for the use of best pain management practices.

Certain identified environmental barriers are not easily modifiable. For example, participants reported shared rooms negatively impact on staff facilitating or involving parents in pain management. Specifically, nursing staff noted insufficient room and concerns about disturbing others in the room, highlighting the importance of single-room maternity care (Stelwagen et al., 2020). However, single-room care for everyone is not currently possible. Given that a core tenet of FCC maternal/newborn care includes non-separation of mother and baby (Public Health Agency of Canada, 2017), adopting a philosophy of 24/7 rooming in is essential, although it requires a cultural and organizational shift, and political will, to transform care into a setting where a philosophy of FCC is truly enacted (Larocque et al., 2021; Ridgway et al., 2021).

Beyond an organizational shift, there is also an important role for governmental and non-governmental organizations and policymakers in supporting practice change. For example, the facilitating effect of two provincial initiatives on the use of the pain management strategies was described. First, the audit and feedback mechanism of the BORN BIS (Better Outcome Registry and Network, 2019b), is a source of positive reinforcement and increased professional confidence. Second, the facilitating effect of working toward Baby-Friendly Hospital Designation (http://www.bfiontario.ca/), as it supports BF and SSC during NBS.

#### The role of laboratory technicians

In this study, some participants reported that lab technicians perform NBS and discussed concerns from lab staff about ergonomics related to blood sampling, scope of practice, and misinformation. However, at one site, education about the study included lab technicians, and their engagement facilitated the use of the pain management strategies. Although there is limited literature regarding the role of lab technicians in newborn pain management, Etchegary et al., (2016) reported that parents and lab technicians expressed that laboratory staff did not use pain reduction strategies during NBS. Piazza et al., (2019) found that phlebotomists infrequently used comfort strategies due in part to a lack of training and education in child development, underscoring the importance of engaging all potential knowledge users when planning for practice change.

#### Ergonomics

One barrier reported both in this study and previously (Benoit et al., 2016; Campbell-Yeo et al., 2017; Harrison et al., 2015; Lavin Venegas et al., 2019b) is the knowledge deficit related to ergonomics of blood sampling while babies are BF or held SSC. To address this knowledge gap, our team had previously partnered with parents, nurses, midwives, and an organizational occupational health physiotherapist to produce a video demonstrating best ergonomics (https://youtu.be/lpZNwP7bnkg). As an intervention that potentially addresses the barrier of ergonomics, an evaluation of the potential usefulness and effectiveness of this publicly accessible video is warranted. While this video was not originally part of the implementation strategy, it was provided to participating units based on their reported barriers.

## Study strengths

The pragmatic choice to use the TDF as a deductive coding matrix is a strength of this study. Employing a theoretical model specifically designed to identify barriers and facilitators to implementation allows for future planning of targeted interventions to address them. A further strength of this study is that we collected feedback from nursing leaders, who are responsible for quality care in their unit and familiar with bedside practice, organizational resources and priorities, initiatives such as the BFI, standards for best practice, and processes at the unit and organizational levels.

## Study limitations

There are two noteworthy limitations of this study. First is the relatively small sample size. Recruitment for this study was challenging (Figure 2), with a lack of response to initial contacts and loss to follow-up during the recruitment processes, primarily due to nursing leadership and staff turnover, and competing priorities. Despite these, data were rich and in-depth, data analysis was rigorous, and findings are transferable to other neonatal settings. Second, although the research team offered monthly telephone interviews and bimonthly CoP teleconferences, and despite email and voicemail reminders from the research coordinator, lack of response from the participants; frequent last-minute cancellations or rescheduling; absenteeism at previously scheduled interviews or CoPs; were challenges. This highlights the difficulty of conducting research in clinical settings.

## Implications for future research

Avenues for future research include studying the paradoxical effect of parent advocacy (that it can simultaneously encourage and discourage certain behaviors), the role of lab technicians in newborn blood sampling, organizational-level contextual barriers, challenges conducting research in the clinical setting, and the effectiveness of dissemination strategies used by our participants.

## Implications for practice

To ensure the consistent use of evidence-based pain management strategies for newborns during routinely performed painful procedures, nursing staff, nursing leadership, and organizational leaders must work in partnership with parents of newborns to facilitate the use of BF, SSC, and sucrose. The parent-targeted educational BSweet2Babies video can be integrated into prenatal education for families; the more recently produced HCP-targeted ergonomics video can be integrated into repeated staff education to help reduce the barrier of lack of comfort with parental presence and perception of high complexity of the strategies.

## Conclusion

This study explored nurse leaders’ perceptions about barriers and facilitators to both showing the BSweet2Babies video and implementing the pain management strategies depicted in the video during NBS. Although the video was considered acceptable and persuasive, most barriers within the TDF domains were identified. Findings highlighted complex multi-level barriers that require support from nursing unit leaders, organizational leadership, and governmental bodies. The facilitating effect of provincial initiatives, such as the BFI and standardized pain treatment data, are promising avenues for future research.

## Supplemental Material

Supplemental Material - Exploring implementation processes of a parent-targeted educational video for improving newborn pain treatment: A sequential exploratory mixed-methods study Supplemental Material for Exploring implementation processes of a parent-targeted educational video for improving newborn pain treatment: A sequential exploratory mixed-methods study by Catherine Larocque, Carolina Lavin Venegas, Sandra Dunn, Marsha Campbell-Yeo, Lucy Gilmore, JoAnn Harrold, Jiale Hu, Leanne McArthur, Shokoufeh Modanloo, Stuart G. Nicholls, Pat O’Flaherty, Shahirose Premji, Jessica Reszel, Sonia Semenic, Janet E. Squires, Bonnie Stevens, Monica Taljaard, Marie-Josee Trepanier, Kathy Venter, Jodi Wilding, and Denise Harrison in Journal of Child Health Care.
