# Supplementary material for: Exploring implementation processes of a parent-targeted educational video for improving newborn pain treatment: A sequential exploratory mixed-methods study
Source: J Child Health Care. 2023 Jun 23;29(1):109–25. doi: 10.1177/13674935231176888 (PMC11874468; doi:10.1177/13674935231176888)
Supplement: Supplemental Material - Exploring implementation processes of a parent-targeted educational video for improving newborn pain treatment: A sequential exploratory mixed-methods study [file sj-pdf-1-chc-10.1177_13674935231176888.pdf]

Supplemental File 1

Parent card

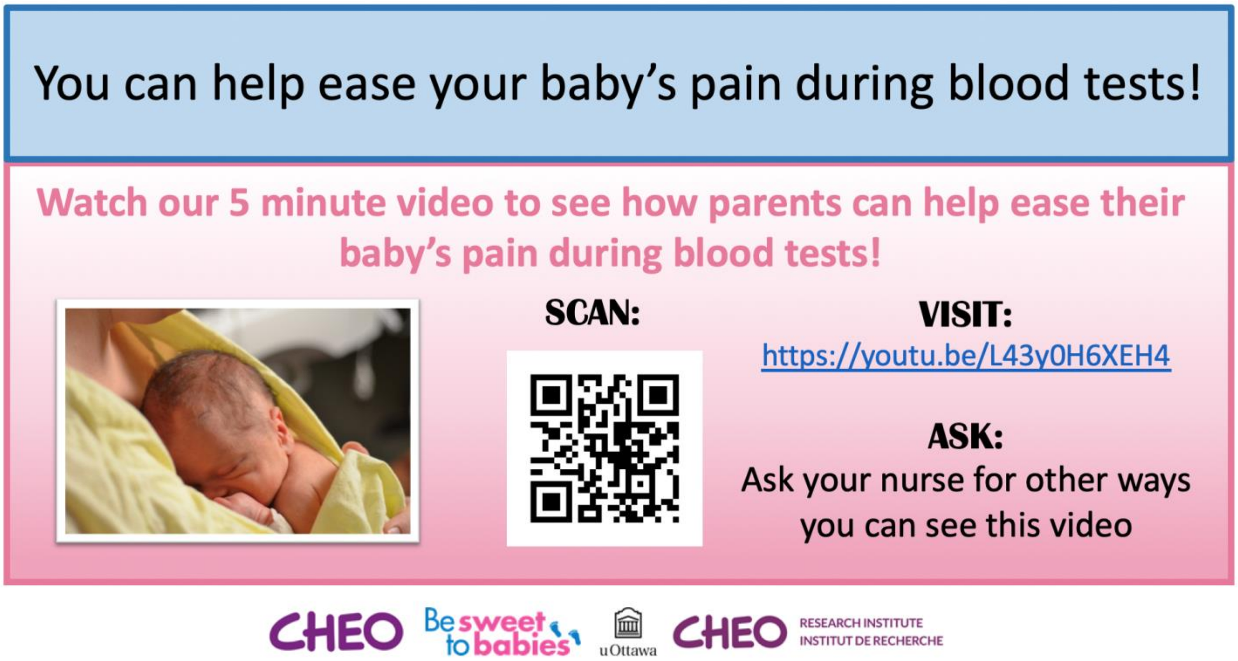

Supplementary File 2

Be Sweet to Babies poster

You can help ease your baby's pain during blood tests!

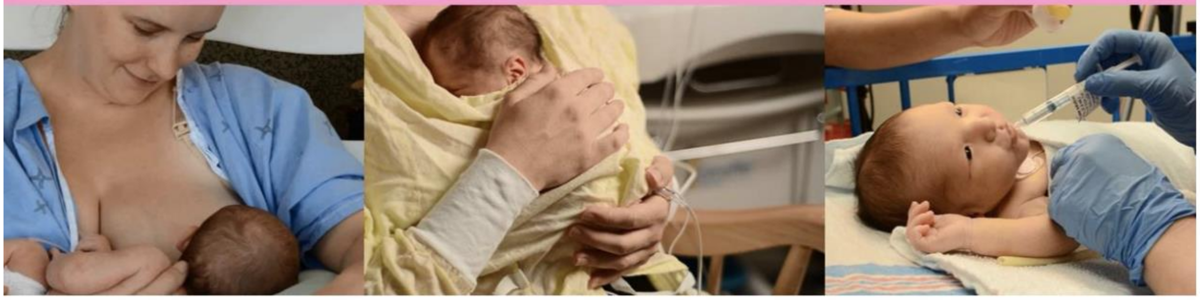

Please **watch our 5-minute video** on the best ways that parents can help ease their baby's pain during newborn screening and other blood tests

SCAN

VISIT

https://tinyurl.com/BeSweet01A

ASK

Ask your nurse for other ways you can see this video

Be sweet to babies

CHEO RESEARCH INSTITUTE INSTITUT DE RECHERCHE

uOttawa

Supplementary File 3

Monthly telephone interview guide

Intervention delivery

Tell me how the last month has gone related to offering and showing parents the BSweet2Babies video.

What proportion of parent are being offered the video? (if not all, why do you think that is?)

What is working well with offering and showing parents the BSweet2babies video?

What are you and/ or your staff finding challenging with offering and showing parents the BSweet2Babies video?

What strategies do you use to optimize the number of parents viewing the video? (e.g., staff education/reminders about showing parents the video)

Is there anything the research team can do to help your site to offer and show the BSweet2Babies video to more/all parents?

Are you noticing an increase in parents advocating for pain management for their newborn's bloodwork?

What is your sense about whether parents or clinicians are initiating the conversation about pain management for newborn bloodwork?

How would you describe your teams (nursing, lab tech etc.) response to the implementation of these pain management strategies in your unit? (*receptive, optimistic, willingness to change, teamwork, flexibility, negative)*

Tell me about the process your organization has used to support this change?

We are interested in strategies to sustain the use of the three pain management strategies that we could share with others sites, do you have any suggestions for such strategies? (Have you implemented these strategies? How is it working?)

Pain treatment data collection processes

Thinking about the entry of the type of pain management data into BORN BIS, can you tell me how the last month has gone?

What is working well with entering the type of pain treatment used during newborn screening into the BIS?

What are you finding challenging with entering the type of pain treatment used during newborn screening into the BIS?

Have you tried any new strategies to increase data entry of pain treatment used during newborn screening

into the BIS?

Is there anything the research team can do to help your site to improve entering the type of pain treatment used during newborn screening into the BIS?

Have you/your unit used the BORN information system to run a report on pain management for your hospital? (Yes or No) If yes, have you provided your staff with the information contained in pain management report? (Yes or No). If yes, what was the outcome?

Supplementary File 4

Facilitators and Barriers for each TDFv2 domain

TDF domain	Domain description[†]	Facilitators	Barriers
Knowledge	Awareness of the existence of something	Being aware of the study taking place and of the three strategies	Lack of awareness of the study/the strategies
Skills	An ability or proficiency acquired through practice		Concerns about positioning and ergonomics<br>Lack of comfort with the technique<br>Lack of comfort with the parents watching
Social professional role and identity	A coherent set of behaviours and displayed personal qualities of an individual in a social or work setting	Professional confidence<br>Unit/Organizational culture that embraces BF/SSC	Scope of practice in a unionized environment
Beliefs about capabilities and Optimism [‡]	Acceptance of the truth, reality, or validity about an ability, talent, or facility that a person	Enthusiasm<br>Belief in the benefits of BF/SSC	

	can put to constructive use; The confidence that things will happen for the best or that desired goals will be attained		
Beliefs about consequences	Acceptance of the truth, reality, or validity about outcomes of a behaviour in a given situation	Holding nurses accountable for performing BF/SSC	Anticipation of parent request (not wanting the parents to ask)
Reinforcement	Increasing the probability of a response by arranging a dependent relationship, or contingency, between the response and a given stimulus	Positive reinforcement from the BIS	
Intentions and Goals‡	A conscious decision to perform a behaviour or a resolve to act in a certain way; Mental representations of outcomes or end states that an individual wants to achieve	Working towards the Baby-Friendly Hospital Initiative designation<br>Participation in the ONesiE study itself<br>Nurses being receptive to changing their practice	Nurses not intending to show the video/perform the strategies

Memory, attention, and decision-making	The ability to retain information, focus selectively on aspects of the environment and choose between two or more alternatives	"Fits" into practice<br>Problem-solving (nurses figuring out how to incorporate video/BF and SSC into their practice)	Competing priorities<br>Forgetfulness
Environmental context and resources	Any circumstance of a person's situation or environment that discourages or encourages the development of skills and abilities, independence, social competence, and adaptive behaviour	Environmental supports<br>Additional staff dedicated to the study<br>Supportive IT departments<br>Support from institution leadership<br>Supporting policies and procedures<br>Access to required equipment (i.e. portable machines nurses can take in the rooms)<br><br>Nature of the intervention ("fits" into practice, various languages, short video)<br><br>KT strategies<br>Posters/pamphlets/cards (to remind nurses and create an empowering environment for parents by allowing them to scan the QR code)<br>Technology (access to TVs and tablets)<br><br>Unit context/factors	Environmental stressors<br>Staffing and staff turnover<br>Unit design (large units are more challenging- the nurses can take the infants to other rooms where the manager and the parents cannot observe them)<br>Special Circumstances (renovations/ implementation of EMR)<br>Lack of supporting policies and documentation<br><br>Technical issues (Wifi, access to Youtube) and lack of technical support<br><br>Nature of the intervention ('unnatural' to show the video, easier face-to-face)<br><br>Unit and OB context<br>Nature of the patients

Single room maternity care (labour/postpartum in the same room)<br>Collaboration with Labour and Delivery units (more time to show the video)<br>Collaboration with pre-registration clinics<br>Smaller units (both size and staff)	(tired after labour)<br>Patient turnover (24 hour stay only)<br>Routines (needing to perform bloodwork overnight)<br>Workload and time constraints

Social influences	Those interpersonal processes that can cause individuals to change their thoughts, feelings, or behaviours	Champions (LC, modified nurse)<br>Group norms and culture (it "becomes" the culture; pressure between colleagues)<br>Parent advocacy (nurses will do it if the parents ask, creates a sense of responsibility)<br>Presence of management (nurses more likely to do blood sampling with baby held BF/SSC and to show video if the manager is present)	Group norms ("hold-outs")
Nature of the behaviour§	What do you do and is that different from what you should do?	Already part of their practice (it is routine)	Way we've always done it (not routine)<br>Already doing the education (video seen as superfluous)
